# The role of red blood cell distribution width in predicting coronary artery lesions in pediatric patients with kawasaki disease

**DOI:** 10.3389/fcvm.2023.1014890

**Published:** 2023-03-03

**Authors:** Jianghui Cai, Mi Tang, Shuping Shuai, Rui Zhang, Hongxi Zhang, Yanfeng Yang, MengJun Wu, Hua Liang, Shasha Xing

**Affiliations:** ^1^ Department of Pharmacy, Chengdu Women's and Children's Central Hospital, School of Medicine, University of Electronic Science and Technology of China, Chengdu, China; ^2^ Office of Good Clinical Practice, Chengdu Women's and Children's Central Hospital, School of Medicine, University of Electronic Science and Technology of China, Chengdu, China; ^3^ Department of Pediatric Cardiology, Chengdu Women's and Children's Central Hospital, School of Medicine, University of Electronic Science and Technology of China, Chengdu, China; ^4^ Department of Anesthesiology, Chengdu Women's and Children's Central Hospital, School of Medicine, University of Electronic Science and Technology of China, Chengdu, China

**Keywords:** kawasaki disease, KD, red blood cell distribution, coronary artery lesion, cals

## Abstract

**Background:**

Recent studies have shown that red blood cell distribution width (RDW) has emerged as a novel predictor of cardiovascular diseases. We aim to investigate the association between RDW and the risk of coronary artery lesions (CALs) in pediatric patients with Kawasaki disease (KD).

**Methods:**

KD patients were classified as the CALs group (patients with CALs) and non-CALs group (patients without CALs). Differences among the groups were analyzed by Mann-Whitney *U*-test and Chi-square analysis. The independent risk factors of CALs were identified by multivariate logistic regression analysis, followed by receiver operating characteristic (ROC) curve analysis to calculate the optimal cut-off value.

**Results:**

The red blood cell distribution width (RDW) and C-reactive protein were significantly higher in the CALs group than those in the non-CALs group (*p* < 0.01). Multivariate logistic regression analysis revealed that RDW (OR = 5.2, 95% CI, 4.064 to 6.654) was independent risk factors of CALs in KD patients (*p* < 0.01). The subgroup analysis also confirmed that the high level of RDW was an independent risk factor for the development of CALs in patients with complete and incomplete KD. The ROC analysis showed the optimal cut-off value of RDW for predicting CALs was >13.86%, with a sensitivity of 75.79% and specificity of 92.81% (AUC = 0.869, 95% CI = 0.844–0.892; *p* < 0.0001).

**Conclusions:**

RDW is an independent predictor with high sensitivity and specificity to predict CALs in KD patients. The elevation in RDW level (>13.86%) may be used as novel biomarkers for early predicting CALs in KD patients during the acute phase.

## Introduction

It has been over half a century since the first Kawasaki disease (KD) case was reported in Japan (1). KD is an acute, self-limited febrile illness with unknown etiology that occurs mainly in children < 5 years of age (2). KD has been a leading cause of acquired heart disease in developed countries because of the increasing rate in recent years (3, 4). The most common complications of KD are coronary artery lesions (CALs) (5), such as dilatation and aneurysm. CALs may persist and progress to obstruction or stenosis, leading to ischemia, myocardial infarction, cardiogenic shock and even sudden cardiac death (6). Thus, the early prediction and prevention of CALs are important to improve outcomes in KD patients, as CALs severely impair the life quality of affected children (7).

Although Intravenous immunoglobulin (IVIG) is an effective medicine to prevent CALs occurrence in KD patients (8), 10%–20% of cases still develop CALs following IVIG application (9). Therefore, it is important to investigate the risk factors of CALs.

Red blood cell distribution width (RDW) is a measure of variation in the size of erythrocytes, which is routinely measured in clinical practice as part of the complete blood count. Currently, studies have provided that RDW had a predictive value in adverse outcomes in KD patients, and RDW was an important risk factor in predicting cardiovascular disease occurrence in the common population (10, 11). However, studies on the predictive value of RDW for CALs in KD patients are limited. Given this background, the current retrospective study aimed to investigate the relationship between RDW and CALs in KD patients and assess the predictive value of RDW for CALs in the acute phase of KD.

## Materials and methods

### Study subjects

This was a retrospective case-control study. We enrolled the KD patients admitted to the Chengdu Women's and Children's Central Hospital aged younger than 18 years old from January 2018 to August 2020. The Chengdu Women's and Children's Central Hospital Ethics Committee approved the study protocol (Approval No.B202212) and waived informed consent requirements. The study conformed to the principles outlined in the Declaration of Helsinki.

The inclusion criteria were as follows: (1) patients were <18 years old; (2) diagnosis of complete or incomplete KD according to the 2017 American Heart Association (AHA) guidelines (2); (3) patients of initial onset of KD; (4) patients received standard treatment with 2 g/kg of IVIG of single infusion during the acute phase of illness. Exclusion criteria were as follows: (1) recurrent KD; (2) history of cardiovascular system diseases; (3) combined with severe infection, allergy, autoimmune diseases, tumor, or blood system diseases; (4) patients who did not receive IVIG or initial IVIG dose was <2 g/kg; (5) patients who had received IVIG in the first three months of admission; (6) receiving drugs that can affect hematological parameters; (7) patients with missing clinical or laboratory information.

### Group assignment

All eligible patients were divided into two groups according to the diagnosis with or without CALs; patients with CALs (CALs group) or patients with non-CALs (non-CALs group). Echocardiography was used to detect CALs during hospitalization. Echocardiography was performed and supervised by the same experienced pediatric cardiologist at our institute. KD patients who met one of the following criteria could be diagnosed as CALs: (1) z score of ≥2 in at least one of the following coronary arteries: right, left anterior descending, and left main according to the AHA guideline in 2017 (2); (2) internal lumen diameter of >2.5 mm in patients <3 years of age, > 3 mm in patients aged 3–9 years, and >3.5 mm in patients aged 9–14 years; (3) internal diameter of a segment measuring ⩾1.5 times that of an adjacent segment; (4) lumen that is clearly irregular (12). The Z-score was measured before IVIG administration using the formula by Dallaire and Dahdah (13).

Complete KD was diagnosed in any children with a fever and had four or more of the following five major symptoms: (I) erythema and cracking of lips, strawberry tongue, and/or erythema of oral and pharyngeal mucosa, (II) polymorphous exanthema, (III) bilateral conjunctival congestion, (IV) changes of the peripheral extremities, and (V) unilateral cervical lymphadenopathy. Incomplete KD was defined as a child with a fever with fewer than four major symptoms and compatible laboratory or echocardiographic findings (2).

### Standard treatment protocol of KD patients

According to our institutional protocol, all KD patients received the same standard therapy with a single 2 g/kg dosage of IVIG and aspirin (30–50 mg/kg/d during the acute phase of illness) immediately after the diagnosis was established. The aspirin was lowered to 3–5 mg/kg/d 2–3 days after the patients were afebrile. Combined antiplatelet and anticoagulation therapy were recommended for patients with giant aneurysms. No additional therapy such as infliximab and plasma exchange was included in the standard treatment protocol. IVIG resistance was defined as recrudescent or persistent fever ≥36 h but not longer than 7 days after the initial IVIG infusion (2). For IVIG-resistant patients, the 2nd IVIG of the same dosage was administrated. If fever persists 36 h after the 2nd IVIG infusion, intravenous methylprednisolone (30 mg/dose) was performed for 3 consecutive days.

### Data collection

Venous blood samples were collected within 24 h pre-IVIG treatment. The blood analysis of the samples was conducted using the Sysmex XN-9,000 automatic blood cell analyzer in the laboratory department of our hospital. All blood test in our hospital has strictly unified procedures for collection, storage, transportation, and examination. The collection of blood samples to the detection results of all specimens is generally no more than 2 h. The demographic, clinical outcomes and laboratory data were extracted from the medical records.

### Statistical analysis

Continuous variables are expressed as mean ± standard deviation or median (InterQuartile Range, IQR) if non-normally distributed. Categorical variables were expressed as numbers and percentages. The normality of the distribution of variables was assessed using the Kolmogorov–Smirnov test. The Chi-square or Fisher's exact test was applied to compare categorical variables. Student's t-test or Mann–Whitney *U*-test was used for continuous variables. Multivariate logistic regression analyses were performed to determine the risk factors of CALs during KD. The receiver operating characteristic curve (ROC) was analyzed to assess the predictive accuracy of RDW for CALs. The sensitivity, specificity, and area under the curve (AUC) were calculated, and the cutoff value was determined by the Youden index. We also performed a subgroup analysis of the types of KD (complete KD and incomplete KD). We estimate that a total of 385 participants will be required for the minimum sample size (14). Statistical significance was defined as a *P* < 0.05. The statistical analyses were performed using SPSS software version 16.0 (SPSS Inc., Chicago, Illinois, United States of America).

## Results

### Characteristics of patients with KD

**Figure 1:**
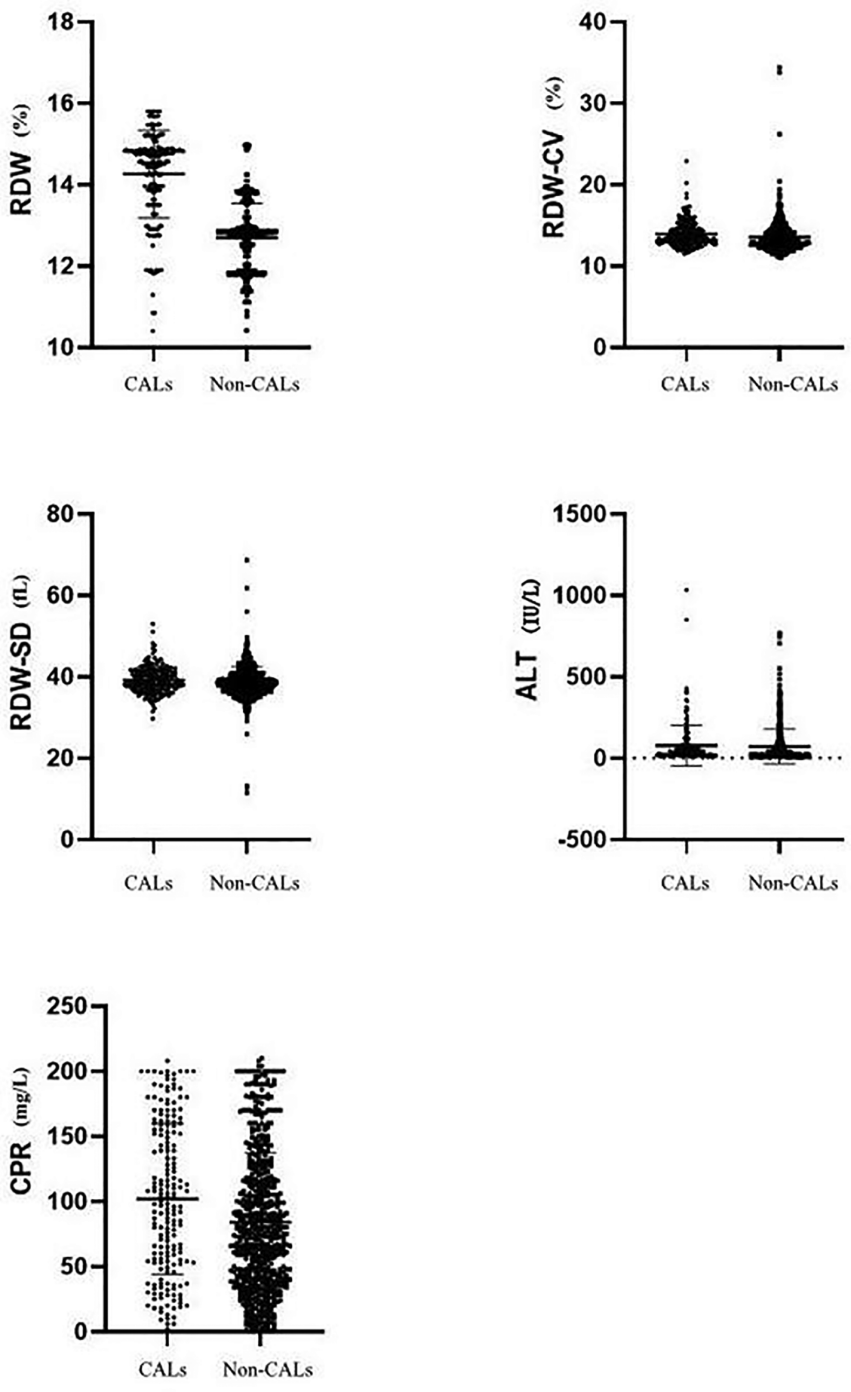
Comparison of RDW, RDW-CV, RDW-SD, ALT, and CRP between CALs group and non-CALs group.

A total of 788 KD patients who meet the inclusion criteria were enrolled in this study (Supplementary Appendix S1), including 516 males and 272 females with ages ranging from 6 to 132 months. Based on echocardiography, 190 patients (24%) were assigned to the CALs group, and 598 (76%) patients were assigned to the non-CALs group. Table 1 shows the differences between the CALs and non-CALs group regarding demographic and laboratory characteristics. There were no significant differences in age, gender, length of illness at initial IVIG treatment, white blood cells (WBC), platelet, neutrophils%, lymphocyte%, counts of lymphocytes, mean corpuscular volume (MCV), erythrocyte sedimentation rate (ESR), and aspartate aminotransferase (AST). Compared with the non-CALs group, the proportions of incomplete KD, the frequency of IVIG resistance, RDW, red blood cell distribution width variation coefficient (RDW-CV), RDW-SD (red blood cell distribution width standard deviation), alanine aminotransferase (ALT), and C-reactive protein (CRP) were significantly higher in patients with CALs (Table 1; Figure 1). In addition, KD patients with CALs had a longer length of hospitalization than KD patients without CALs. On the contrary, haemoglobin (HB) was significantly lower in patients with CALs.

**Table 1:** Demographic and laboratory characteristics of patients with kawasaki disease.

	CAL group (*N* = 190)	Non-CAL group (*N* = 598)	*U* or *χ^2^*	*P*
**Age (Months)**, Median (IQR)	20 (8.00∼42.50)	19 (10.50∼34.50)	−1.479	0.139
**Gender**, *n* (%)			1.765	0.184
Male	132 (69.47)	384 (64.21)		
Female	58 (30.53)	214 (35.79)		
**Diagnosis**, *n* (%)			14.709	<0.001
Complete	161 (84.74)	560 (93.65)		
Incomplete	29 (15.26)	38 (6.35)		
**Length of illness at initial IVIG treatment (day)**, Median (IQR)	6.00 (5.00∼7.00)	6.00 (5.00∼7.00)	−1.888	0.059
**Length of hospitalization (days)**, Median (IQR)	8.00 (6.00∼9.00)	7.00 (6.00∼9.00)	−3.39	0.001
**IVIG resistance**, *n* (%)			9.396	0.002
Yes	31 (16.32)	50 (8.36)		
No	159 (83.68)	548 (91.64)		
**WBC count ( × 10^9^/L)**, Median (IQR)	14.06 (10.83∼17.82)	13.56 (10.485∼17.26)	−0.432	0.666
**HB (g/L)**, Median (IQR)	107.00 (98.00∼113.50)	107.00 (99.00∼115.50)	−2.001	0.045
**Platelet ( × 10^9^/L)**, Median (IQR)	355.00 (274.50∼444.50)	356.00 (271.75∼428.25)	−0.991	0.322
**Neutrophils (%)**, Median (IQR)	66.00 (54.15∼76.20)	66.40 (55.70∼78.43)	−0.422	0.673
**Lymphocyte (%)**, Median (IQR)	23.60 (14.95∼34.20)	24.45 (14.95∼32.93)	−0.606	0.545
**Lymphocyte count**	3.22 (1.98∼4.74)	3.17 (1.94∼4.43)	−0.746	0.455
**MCV (fL)**	80.80 (77.65∼84.25)	81.70 (79.05∼84.00)	−1.203	0.229
**RDW (%)**	14.58 (13.87∼14.86)	12.78 (12.00∼13.20)	−15.356	<0.001
**RDW-CV (%)**	13.60 (12.90∼14.65)	13.10 (12.5∼14.1)	−4.28	<0.001
**RDW-SD (fL)**	38.70 (36.90∼41.25)	38.30 (36.35∼40.3)	−2.192	0.028
**CRP (mg/L)**, Median (IQR)	98.00 (53.50∼155.00)	72.00 (36.50∼117.50)	−3.667	<0.001
**ESR (mm/h)**, Median (IQR)	72.00 (50.00∼90.00)	72.00 (45.00∼90.00)	−0.354	0.723
**ALT (IU/L)**, Median (IQR)	35.20 (19.65∼70.65)	32.20 (15.20∼83.20)	−2.146	0.032
**AST (IU/L)**, Median (IQR)	35.90 (25.85∼52.80)	32.90 (24.70∼54.18)	−1.11	0.267

IVIG, intravenous immunoglobulin; WBC, white blood cell count; HB, haemoglobin; MCV, mean corpuscular volume; RDW, red blood cell distribution width; RDW-CV, ed blood cell distribution width variation coefficient; RDW-SD, red blood cell distribution width standard deviation; CRP, C-reactive protein; ESR, erythrocyte sedimentation rate; ALT, alanine aminotransferase; AST, aspartate aminotransferase.

### Characteristics of patients with complete kd and incomplete KD

A total of 721 children were diagnosed with complete KD (91.5%), and 161 had CALs. Sixty-seven patients (8.5%) had incomplete KD, and 29 children with KD had CALs. In the subgroup analysis of complete KD, compared with the group without CALs, length of hospitalization, the frequency of IVIG resistance, RDW, RDW-CV, RDW-SD, CRP, and ALT were significantly higher in patients with CALs (Supplementary Appendix S2). In the incomplete KD subgroup, only RDW was significantly higher in patients with CALs than in those without CALs (Supplementary Appendix S3).

### Independent risks for predicting CALs by multivariate regression analysis

Next, because the length of hospitalization, IVIG resistance, HB, RDW, RDW-CV, RDW-SD, ALT, and CRP all showed significant differences (*P* < 0.05) between the two groups, they were chosen as independent variables. Using the occurrence of CALs as the dependent variable, we performed a multivariate logistic regression analysis to evaluate the independent risks for predicting CALs (Supplementary Appendix S4). The results indicated that RDW (OR = 5.2, 95% CI, 4.064 to 6.654) was independent risk factors of CALs in KD patients (*p* < 0.01). In addition, multivariate regression analysis also showed that the high RDW level was an independent risk factor for the development of CALs in patients with complete KD and incomplete KD (Table 2).

**Table 2:** Multivariate logistic regression analysis for risk factors of CALs in KD patients (including complete and incomplete KD).

	B	S.E.	Wald	*P*	OR	95% C.I. of OR	
						Lower	Upper
**KD patients (including complete and incomplete KD)**							
RDW	1.649	0.126	171.752	<0.001	5.2	4.064	6.654
**Complete KD**							
Length of hospitalization (days)	0.047	0.053	0.809	0.368	1.048	0.946	1.162
IVIG resistance	−0.547	0.341	2.567	0.109	0.579	0.297	1.13
RDW	1.642	0.133	153.186	<0.001	5.165	3.983	6.699
RDW-CV	−0.01	0.045	0.051	0.822	0.99	0.907	1.081
RDW-SD	0.007	0.032	0.05	0.822	1.007	0.945	1.073
CRP	0.004	0.002	3.16	0.075	1.004	1	1.008
ALT	−0.001	0.001	0.839	0.36	0.999	0.997	1.001
**Incomplete KD**							
RDW	1.702	0.389	19.114	<0.001	5.486	2.558	11.766

IVIG, intravenous immunoglobulin; RDW, red blood cell distribution width; RDW-CV, Red blood cell distribution width variation coefficient; RDW-SD, Red blood cell distribution width standard deviation; CRP, C-reactive protein; ALT, alanine aminotransferase.

### ROC analysis

**Figure 2:**
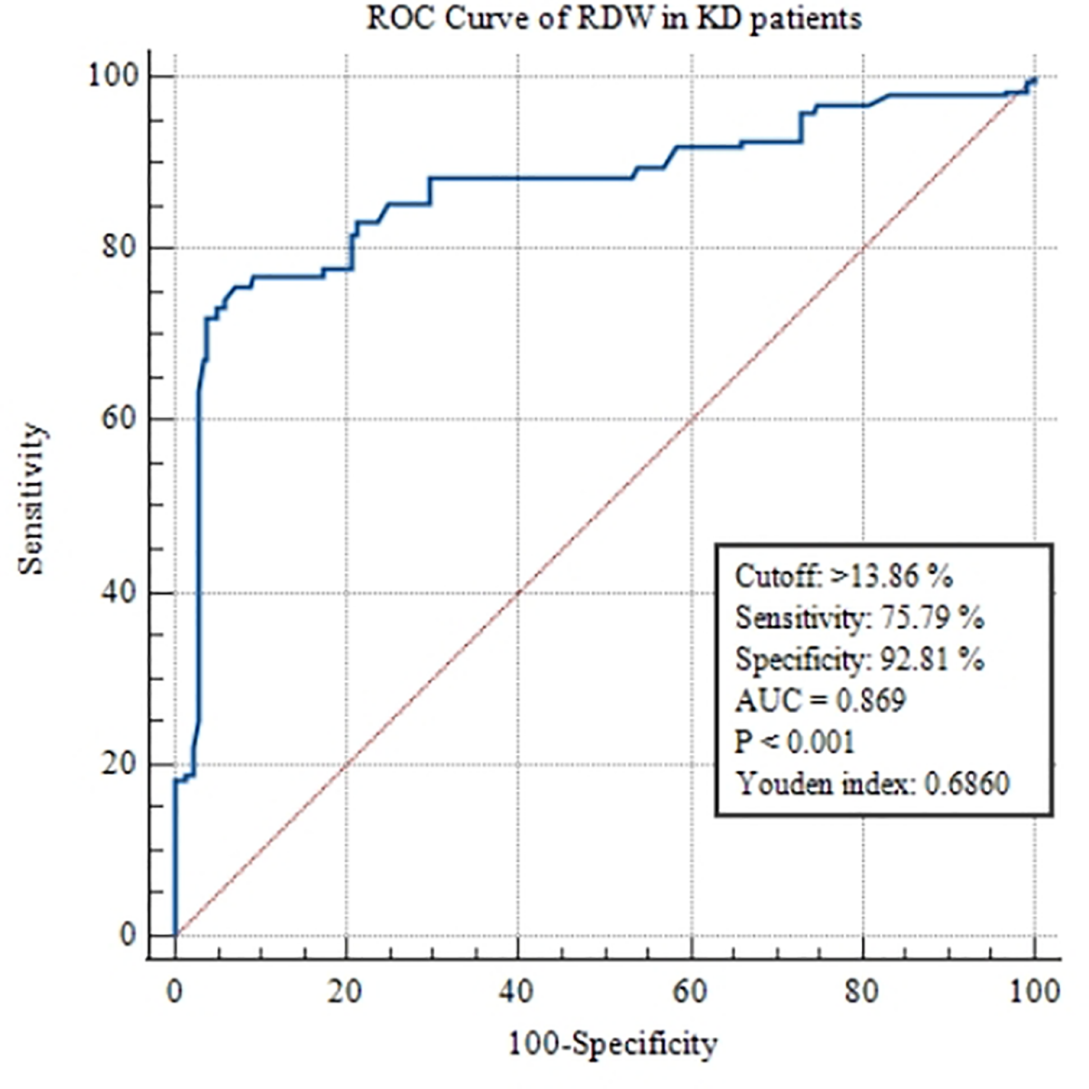
ROC curve analysis of RDW to predict CALs in KD patients.

**Figure 3:**
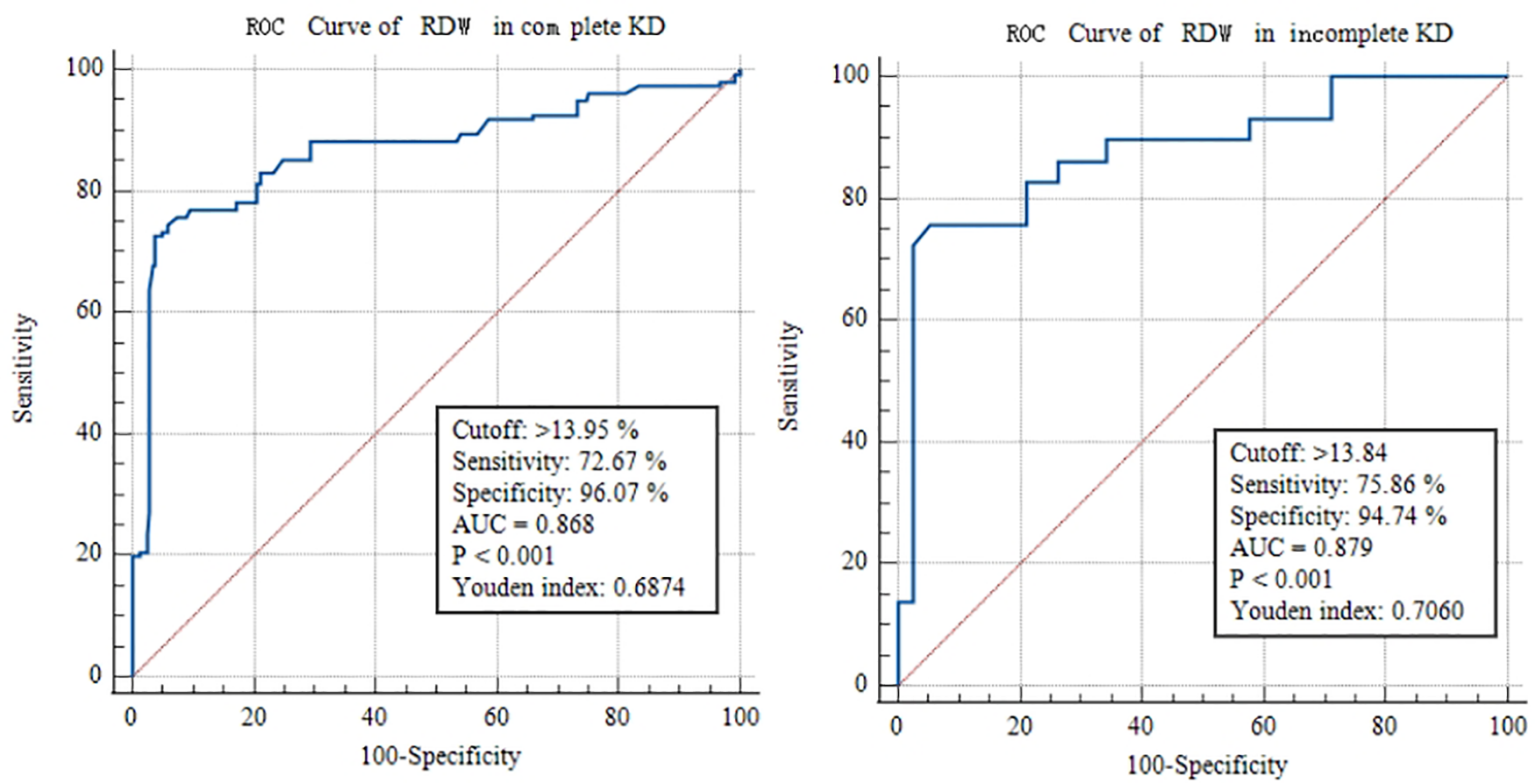
ROC curve analysis of RDW to predict CALs in complete and incomplete KD patients.

According to the ROC analysis, the optimal cut-off value of RDW in KD patients for predicting CALs was >13.86%, with a sensitivity of 75.79% and a specificity of 92.81% (AUC was 0.869, 95% confidence interval 0.844–0.892; *p* < 0.0001; Figure 2). The optimal cut-off value of RDW in patients with complete KD for predicting CALs was >13.95%, with a sensitivity of 72.67% and a specificity of 96.07% (AUC was 0.868, 95% confidence interval 0.841–0.892; *p* < 0.0001; Figure 3). On the other hand, the ROC of RDW in incomplete KD patients showed that the optimal cut-off value, sensitivity, and specificity were >13.84%, 75.86%, and 94.74%, respectively (AUC was 0.879, 95% confidence interval 0.776–0.946; *p* < 0.0001; Figure 3).

### Discussion

The present study aimed to investigate the association between RDW and the risk of CALs in pediatric patients with KD. Our study showed that RDW was significantly higher in the CALs group than in the non-CALs group. Multivariate regression analysis revealed that a high level of RDW (>13.86%) on admission was an independent risk (OR=5.2, 95% CI, 4.064 to 6.654) for predicting CALs in KD patients. The AUC of RDW for the predictor of CALs in KD patients was 0.869 (95% confidence interval 0.844–0.892; *p* < 0.0001), with a sensitivity and a specificity value of 75.79% and 92.81%, respectively. Thus, RDW may be helpful for predicting CALs in the acute phase of KD.

Although risk factors for CALs in KD have been studied extensively, there are no reliable indicators for predicting CALs in clinical practice have been established thus far. On the other hand, previous studies that attempted to predict the risk factors for developing CALs in children with KD reported discrepant results (15–18). RDW is an index that primarily reflects impaired erythropoiesis and abnormal red blood cell survival (19). RDW is routinely measured in clinical practice as part of the complete blood count and is commonly used to diagnose anemia. The interest in the clinical value of RDW has been growing since numerous studies have demonstrated that a high level of RDW is an independent predictor of morbidity in cardiovascular diseases (20, 21). Wang et al. (22) measured RDW in 1,654 patients with the acute coronary syndrome (ACS). Higher RDW was associated with increased 1-month heart failure and recurrent infarction. Similarly, Cemin et al. (23) reported RDW was found to be a significant predictor of ACS in 1,971 consecutive patients. The RDW values were also significantly increased in patients with coronary artery disease than those without and were significantly higher in patients with more severe coronary artery disease (23). Nevertheless, studies regarding the relationship between RDW and CALs in pediatric patients with KD are scarce (24, 25).

The level of RDW is generally elevated due to ineffective RBC production, increased RBC destruction, or blood transfusion. There are several possible explanations, although the mechanism of the relationship between increased RDW and the development of CALs in KD patients remains unknown. The inflammatory reaction is one of the possible mechanisms and plays a critical role in developing CALs. It is known that KD is an auto-immune disorder reported to be caused by an over-activated immune response to pathogen infections and produces excessive inflammatory cytokines, eventually leading to immune vasculitis (26). RDW may be an integrative marker, reflecting multiple biological imbalances contributing to higher coronary artery lesion risks in KD patients, such as chronic inflammation and oxidative stress. RDW may have a role in inflammation activation, triggered a culmination of multiple pathophysiologic processes, and then resulted in CALs. Thus, we speculated that inflammation may bridge the relationship between elevated RDW and KD prognosis although further multicenter studies are needed to determine the potential mechanisms linking RDW and prognosis. Lippi et al. (27) found a correlation between a high level of RDW and elevated indices of inflammation, such as C-reactive protein levels. This correlation was independent of concomitant diseases and was also demonstrated in anaemic patients (27). In the present study, RDW and CRP in the CALs group were significantly higher than the levels in non-CALs group, showing consistency with previous studies (28). These results implicated that elevated RDW on admission indicates a severe inflammatory process, which may be an early indicator of CALs in KD patients. Furthermore, it is worth noting that multivariate logistic regression analysis showed RDW as a risk factor of CALs in KD patients with a large effect (OR=5.2), which indicates RDW may be more effective in predicting the prognosis of KD than other cardiovascular diseases (29).

In addition, a previous study showed that a high level of hepcidin (indicators of iron deficiency anemia) leads to a low serum iron content and limits the availability of iron for erythropoiesis, resulting in an increase in RDW (30). Kuo et al. (31) also reported that hepcidin is associated with anemia development and clinical outcomes in KD patients. However, we could not confirm a relationship between RDW and iron metabolism because no indices reflecting iron metabolism were available in our study. We found that KD patients with CALs had a lower mean erythrocyte volume than those without CALs, which may indirectly reflect iron deficiency, although it was not statistically significant (*p* = 0.229). Thus, impaired iron mobilization may be a pathophysiological mechanism of the association between increased RDW and CALs.

RDW can be expressed as RDW-CV and RDW-SD. RDW-SD has better sensitivity and is less affected by other factors than RDW-CV (32). Nevertheless, studies so far only focus on RDW-CV as a predictor of CALs in patients with KD. In the present study, neither RDW-CV nor RDW-SD is an independent risk factor for CALs in KD patients. Our findings call for future studies on the prognostic value of RDW should focus on RDW-SD to better understand the mechanism by which RDW relates to KD. Multivariate regression revealed that the high proportion of incomplete KD is the other independent risk factor of CALs, which is compatible with the previous studies (33, 34).

### Limitations

The present study has several limitations. First, this study was designed as retrospective in nature, which means potential selection or information bias may exist. Second, all the KD patients came from Sichuan, southwest China, limiting the findings' generalization. Therefore, a future prospective multicenter study is needed to verify the present results.

## Conclusions

RDW is an independent predictor (OR = 5.2, 95% CI, 4.064 to 6.654) with high sensitivity and specificity to predict CALs in KD patients. The elevation in RDW level (>13.86%) may be used as novel biomarkers for early predicting CALs in KD patients during the acute phase. However, further prospective multicenter studies are required to verify the predictive value of RDW for CALs in KD patients.

## Data Availability

The original contributions presented in the study are included in the article/**Supplementary Material**, further inquiries can be directed to the corresponding author/s.
