# Supplementary material for: The role of red blood cell distribution width in predicting coronary artery lesions in pediatric patients with kawasaki disease
Source: Front Cardiovasc Med. 2023 Mar 3;10:1014890. doi: 10.3389/fcvm.2023.1014890 (PMC10020711; doi:10.3389/fcvm.2023.1014890)
Supplement: Supplementary file 1 [file Datasheet1.docx]

**Contents of supplementary materials :**

**1) The flowchart of the retrospective study;**

**2) Baseline characteristics of the complete KD patients;**

**3) Baseline characteristics of the incomplete KD patients;**

**4) Multivariate logistic regression analysis for risk factors of CALs in KD patients.**

**Appendix S1. The flowchart of the retrospective study.**


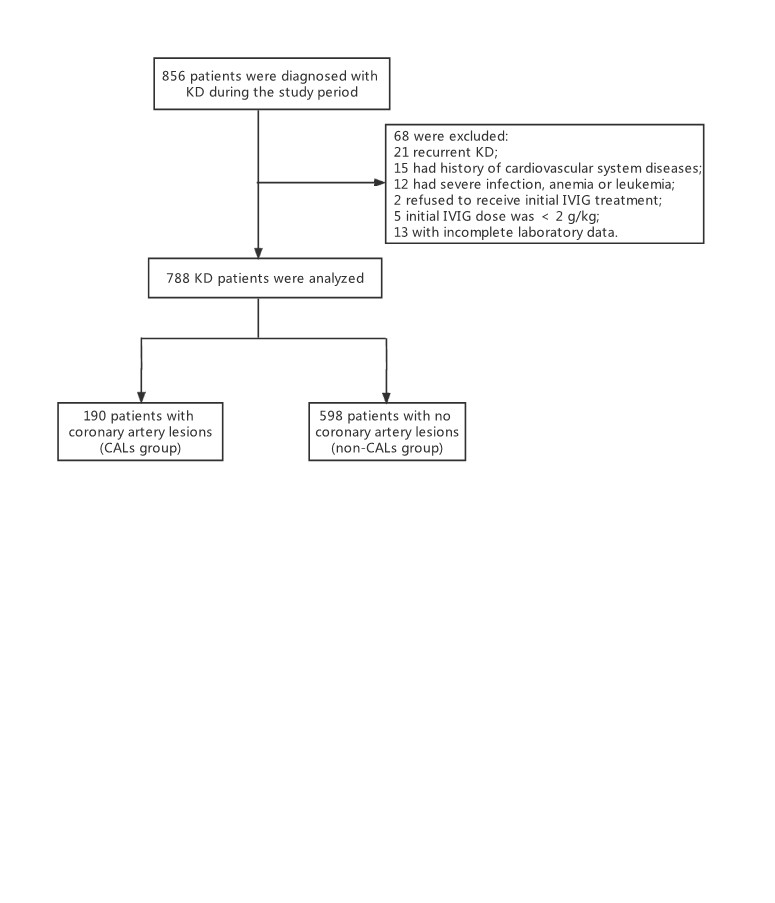


Note: CALs = coronary artery lesions.

**Appendix S2. Baseline characteristics of the complete KD patients.**

|  | **CAL group**  **(N=161)** | **Non-CAL group**  **(N=560)** | ***U* or *χ^2^*** | ***P*** |
| --- | --- | --- | --- | --- |
| **Age (Months)**, Median (IQR) | 20 (9~43) | 20 (11~35) | -0.865 | 0.387 |
| **Gender**, n (%) |  |  | 1.11 | 0.292 |
| Male | 111 (68.94) | 361 (64.46) |  |  |
| Female | 50 (31.06) | 199 (35.54) |  |  |
| **Length of illness at initial IVIG treatment (day)**, Median (IQR) | 6.00 (5~7) | 6.00 (5~7) | -0.93 | 0.352 |
| **Length of hospitalization (day)**, Median (IQR) | 8 (6~9) | 7 (6~9) | -2.912 | 0.004 |
| **IVIG resistance**, n (%) |  |  | 6.637 | 0.01 |
| Yes | 28 (17.39) | 56 (10) |  |  |
| No | 133 (82.61) | 504 (90) |  |  |
| **WBC count (×10^9^/L)**, Median (IQR) | 14.22 (10.80~17.66) | 13.30 (10.48~17.26) | -0.475 | 0.635 |
| **HB (g/L)**, Median (IQR) | 108.00 (99.25~113.50) | 107.00 (99.00~115.00) | -1.443 | 0.149 |
| **Platelet (×10^9^/L)**, Median (IQR) | 348.50 (269.50~431.75) | 354.00 (274.75~446.75) | -0.453 | 0.65 |
| **Neutrophils (%)**, Median (IQR) | 67.60 (57.48~78.24) | 67.05 (55.17~78.58) | -0.392 | 0.695 |
| **Lymphocyte (%)**, Median (IQR) | 22.00 (14.20~33.28) | 23.20 (14.73~33.43) | -0.088 | 0.93 |
| **Lymphocyte count** | 3.05 (1.91~4.71) | 3.14 (1.91~4.52) | -0.194 | 0.846 |
| **MCV (fL)** | 80.90 (77.93~84.38) | 81.80 (79.15~84.15) | -0.589 | 0.556 |
| **RDW (%)** | 14.71  (13.87~14.86) | 12.78  (11.94~13.33) | -14.243 | <0.001 |
| **RDW-CV (%)** | 13.55  (12.90~14.60) | 13.10  (12.50~13.98) | -3.763 | <0.001 |
| **RDW-SD (fL)** | 38.75  (36.90~41.20) | 38.20  (36.30~40.45) | -2.027 | 0.043 |
| **CRP (mg/L)**, Median (IQR) | 106.00 (54.50~160.00) | 72.00 (38.00~116.00) | -3.774 | <0.001 |
| **ESR (mm/h)**, Median (IQR) | 72.50 (50.00~91.00) | 72.50 (47.00~89.00) | -0.043 | 0.966 |
| **ALT (IU/L)**, Median (IQR) | 35.20 (19.73~78.78) | 31.65  (15.75~99.53) | -2.374 | 0.018 |
| **AST (IU/L)**, Median (IQR) | 36.00 (25.93~54.25) | 33.25 (25.15~57.88) | -1.063 | 0.288 |

IVIG = intravenous immunoglobulin; WBC= white blood cell count; HB=haemoglobin; MCV = Mean Corpuscular Volume; RDW =red blood cell distribution width; RDW-CV = Red blood cell distribution width variation coefficient; RDW-SD = Red blood cell distribution width standard deviation; CRP = C-reactive protein; ESR = erythrocyte sedimentation rate; ALT = alanine aminotransferase; AST = aspartate aminotransferase.

**Appendix S3. Baseline characteristics of the incomplete KD patients.**

|  | **CAL group**  **(N=29)** | **Non-CAL group**  **(N=38)** | ***U* or *χ^2^*** | ***P*** |
| --- | --- | --- | --- | --- |
| **Age (Months)**, Median (IQR) | 12 (4~41) | 23 (11~39) | -1.33 | 0.184 |
| **Gender**, n (%) |  |  | 1.031 | 0.31 |
| Male | 21 (69.47) | 23 (60.53) |  |  |
| Female | 8 (30.53) | 15 (39.47) |  |  |
| **Length of illness at initial IVIG treatment (day)**, Median (IQR) | 8 (6~10) | 8 (7~10) | -0.384 | 0.701 |
| **Length of hospitalization (day)**, Median (IQR) | 8 (7~9) | 7 (6~9) | -0.716 | 0.474 |
| **IVIG resistance**, n (%) |  |  | 0.001 | 0.981 |
| Yes | 3 (10.34) | 4 (10.53) |  |  |
| No | 26 (89.66) | 34 (89.47) |  |  |
| **WBC count (×10^9^/L)** | 13.74 ± 5.19 | 13.28 ± 4.81 | 0.376 | 0.708 |
| **HB (g/L)** | 101.14 ± 14.04 | 103.45 ± 12.72 | -0.704 | 0.484 |
| **Platelet (×10^9^/L)** | 443.59 ± 185.27 | 415.71 ± 149.24 | 0.682 | 0.498 |
| **Neutrophils (%)** | 58.51±12.28 | 59.04±18.04 | -0.135 | 0.893 |
| **Lymphocyte (%)** | 30.38±11.52 | 28.59±13.87 | 0.562 | 0.576 |
| **Lymphocyte count** | 3.81 (2.29~5.19) | 3.41 (1.94~4.51) | -1.19 | 0.234 |
| **MCV (fL)** | 79.80 (75.40~84.25) | 82.30 (79.50~84.40) | -1.588 | 0.112 |
| **RDW (%)** | 14.52  (13.69~14.78) | 12.84  (12.25~13.18) | -5.285 | <0.001 |
| **RDW-CV (%)** | 13.80  (12.90~15.95) | 13.80  (12.80~14.55) | -1.526 | 0.127 |
| **RDW-SD (fL)** | 38.80  (37.10~41.85) | 38.60  (36.85~41.15) | -0.411 | 0.681 |
| **CRP (mg/L)**, Median (IQR) | 82.00 (46.00~114.50) | 49.00 (38.00~101.50) | -1.152 | 0.249 |
| **ESR (mm/h)**, Median (IQR) | 69.00 (41.50~88.00) | 82.00 (49.50~100.50) | -0.905 | 0.365 |
| **ALT (IU/L)**, Median (IQR) | 26.50 (17.35~48.05) | 19.90  (17.95~63.30) | -0.664 | 0.506 |
| **AST (IU/L)**, Median (IQR) | 34.60 (25.60~46.20) | 30.30 (24.30~53.25) | -0.633 | 0.527 |

IVIG = intravenous immunoglobulin; WBC= white blood cell count; HB=haemoglobin; MCV = Mean Corpuscular Volume; RDW =red blood cell distribution width; RDW-CV = Red blood cell distribution width variation coefficient; RDW-SD = Red blood cell distribution width standard deviation; CRP = C-reactive protein; ESR = erythrocyte sedimentation rate; ALT = alanine aminotransferase; AST = aspartate aminotransferase.

**Appendix S4. Multivariate logistic regression analysis for risk factors of CALs in KD patients.**

|  | **B** | **S.E.** | **Wald** | **P** | **OR** | **95%C.I. of OR** | |
| --- | --- | --- | --- | --- | --- | --- | --- |
|  |  |  |  |  |  | **Lower** | **Upper** |
| Length of hospitalization (days) | 0.052 | 0.051 | 1.043 | 0.307 | 1.054 | 0.953 | 1.165 |
| IVIG resistance (No vs. Yes) | -0.431 | 0.323 | 1.778 | 0.182 | 0.65 | 0.345 | 1.225 |
| HB | -0.002 | 0.009 | 0.052 | 0.819 | 0.998 | 0.98 | 1.016 |
| RDW | 1.649 | 0.126 | 171.752 | <0.001 | 5.2 | 4.064 | 6.654 |
| RDW-CV | 0 | 0.03 | 0 | 0.989 | 1 | 0.943 | 1.059 |
| RDW-SD | 0.001 | 0.03 | 0.001 | 0.98 | 1.001 | 0.944 | 1.061 |
| CRP | 0.004 | 0.002 | 3.265 | 0.071 | 1.004 | 1 | 1.008 |
| ALT | -0.001 | 0.001 | 1.369 | 0.242 | 0.999 | 0.997 | 1.001 |

IVIG = intravenous immunoglobulin; HB = haemoglobin; RDW = red blood cell distribution width; RDW-CV = red blood cell distribution width variation coefficient; RDW-SD = red blood cell distribution width standard deviation; CRP = C-reactive protein; ALT = alanine aminotransferase.
